# Long-term space missions’ effects on the human organism: what we do know and what requires further research

**DOI:** 10.3389/fphys.2024.1284644

**Published:** 2024-02-13

**Authors:** Marcin Tomsia, Julia Cieśla, Joanna Śmieszek, Szymon Florek, Agata Macionga, Katarzyna Michalczyk, Dominika Stygar

**Affiliations:** ^1^ Department of Forensic Medicine and Forensic Toxicology, Faculty of Medical Sciences in Katowice, Medical University of Silesia, Katowice, Poland; ^2^ School of Medicine in Katowice, Medical University of Silesia, Katowice, Poland; ^3^ Department of Physiology, Faculty of Medical Sciences in Zabrze, Medical University of Silesia, Katowice, Poland; ^4^ SLU University Animal Hospital, Swedish University of Agricultural Sciences, Uppsala, Sweden

**Keywords:** astronauts, cosmic radiation, long-term space mission, microgravity, space physiology

## Abstract

Space has always fascinated people. Many years have passed since the first spaceflight, and in addition to the enormous technological progress, the level of understanding of human physiology in space is also increasing. The presented paper aims to summarize the recent research findings on the influence of the space environment (microgravity, pressure differences, cosmic radiation, etc.) on the human body systems during short-term and long-term space missions. The review also presents the biggest challenges and problems that must be solved in order to extend safely the time of human stay in space. In the era of increasing engineering capabilities, plans to colonize other planets, and the growing interest in commercial space flights, the most topical issues of modern medicine seems to be understanding the effects of long-term stay in space, and finding solutions to minimize the harmful effects of the space environment on the human body.

## 1 Introduction

The main factors that change the functioning of the human organism in space are microgravity (understood as conditions with minimal gravitational acceleration) and exposure to cosmic radiation. Cosmic radiation consists of galactic cosmic rays (GCR) and solar particle events (SPE). The GCR comprises high-energy protons, helium nuclei, and heavy nuclei with energies above 10 GeV/amu. Heavy ions, which account for only 1% of the total mass, belong to high linear energy transfer (LET) radiation, which can seriously damage human cells, tissues, and organs. Shielding against GCR is very difficult, and ordinary physical shielding methods cannot achieve effectively protect astronauts (Guo et al., 2022). Whereas SPEs are mostly protons with energies ranging from 10 MeV to 100 MeV. Highly energetic SPEs can also produce near relativistic protons in a magnitude of 20 GeV (NCRP Report No, 2000; Marusek, 2007). The Earth’s average annual natural radiation exposure is approximately 2.4 millisieverts (mSv) (United Nations, 2023b). International Space Station’s (ISS) 6 months exposure is defined by a dose equivalent to 50–100 mSv, and Mars Mission exposure is estimated between 870 and 1,200 mSv (Simonsen et al., 2020). To illustrate this, the ISS mission equals to ∼53 head computed tomography (CT), and the Mars Mission to ∼632 head CT or 12,000 posterior-anterior (PA) adult chest X-rays, where a single head CT and X-ray is 1.9 mSv and 0.1 mSv, respectively (Pantos et al., 2011; CAR, 2012).

Experiments with terrestrial animal models exposed to cosmic rays or simulated microgravity conditions are used to assess health risks of long interplanetary journeys and develop potential countermeasures protecting human bodies. Data from shorter spaceflights inform about possible reaction of the human body to the space environment, which become more likely the longer the flight takes (Makedonas et al., 2018). Although the presented review mainly focuses on the effects of long-term spaceflights, the short-term spaceflight data are also included due to their high scientific value and the limited number of long-term spaceflight studies.

Due to the rapid progress of space engineering and technology, and the growing popularity of commercial space flights, it seems crucial to thoroughly study the impact of the space environment on the human body. Knowledge about the type of adaptation of individual tissues and organs to the space environment would allow to develop methods preventing the adverse effects of long-term stay in space, which is necessary for future progress of space technology. The need to study the impact of long-term space flights on the human body is strong as new countermeasures, to deal with adverse health events occurring during and after a space mission, are being developed. In-depth risk analysis is a key element of this process. It involves identifying large hazards for space flights based on the elements of space missions: destination, duration, gravitational and radiation environment, etc. (Romero and Francisco, 2020). NASA’s human risk management is based on specialized protocols that determine the risk associated with specific threats for each human body part depending on the risk associated with external factors (Antonsen et al., 2023). The importance of individual elements of “the big 5 hazards” on the human body is discussed in individual chapters.

The review aims to present the greatest challenges for the human body related to long-term spaceflight. The review summarizes the most important risk factors related to long-term stay in space focusing on human cardiovascular system, respiratory system, nervous system and specialized senses, musculoskeletal system, digestive system, excretory system, immune response, and hormonal and reproductive functions. The review focuses on research from recent years (2018–2023), referring also to the discoveries from the beginning of the history of spaceflights. The review also presents the biggest challenges and problems that must be solved before the time of human stay in space is safely extended.

## 2 Cardiovascular system

The adverse impact of space environment on the cardiovascular system consists mainly of reduced exercise tolerance and physical fitness. However, the greatest threats to the human body are orthostatic intolerance and altered heart electrical rhythm resulting from the changed gravity field (Romero and Francisco, 2020). During spaceflight, circulating blood volume decreases significantly, resulting in decreased stroke volume and blood pressure and a compensatory heart rate increase. It is estimated that the astronaut’s exercise tolerance equals to the efficiency of a sedentary person (Convertino, 2005; Gallo et al., 2020).

Analysis of the heart rate and blood pressure during spaceflights and state of weightlessness indicates that the vascular system adapts to these conditions by relaxing. Staying in space for up to 6 months causes a decrease in heart rate (HR), arterial pressure (BP), and pulse pressure (PP) (Verheyden et al., 2009) and the changes are progressive. For an astronaut in space, the heart rate decreased by an 176 ms interval, which translated to a 20% decrease in HR. In this study the initial adaptation took place after the first month of the mission (HR decreased by 82 ms) and deepened during the 6 months of the space mission by another 94 ms. A longer stay in space did not further change the duration of the interval between heartbeats (Gundel et al., 2002). These changes are crucial for people returning from space to Earth conditions who experience orthostatic intolerance upon return. Orthostatic intolerance depends on reflexes and neuronal regulation and is a huge problem not only for astronauts returning from spaceflights, but also for people after a long-term hospitalization that required lying position for a long time (Blaber et al., 2022). According to Sigaudo-Roussel et al., the astronauts who fail to stand for 5 min in the first days after spaceflight present lower systolic blood pressure than those who are able to complete this test. This phenomenon results from impaired baroreceptor function and blood pressure control (Sigaudo-Roussel et al., 2002). Orthostatic hypotension occurring after spaceflight more often affects women and astronauts who have low vascular resistance and a predominant hypoadrenergic response (Waters et al., 2002). It is believed that orthostatic disorders might relate to the dysfunction of the vestibular otoliths, which are involved in maintaining balance (Hallgren et al., 2015; Hallgren et al., 2016). Many studies of post-flight hypotension were conducted standing or using a tilt table, while Fu et al. examined the circulatory parameters of astronauts during daily activities. None of the 12 analyzed subjects experienced orthostatic intolerance after a 6-month space mission. Systolic blood pressure returned to normal shortly after return to Earth, more specifically—the landing day. The results proved the effectiveness of the applied prophylactic methods: in-flight exercise training and volume resuscitation on return (Fu et al., 2019). However, the contradictory results of research on blood pressure and HR parameters, indicate that it is necessary to constantly improve prophylactic methods to minimize blood pressure disorders in people returning from space.

The variability of the heart rate during a long-term stay in space is a valuable indicator of human body adaptation to long-term stay in the state of microgravity (Otsuka et al., 2018). A prolonged stay in space induces the variability of the sleep and wakefulness rhythm and changes in the autonomic system activity. During wakefulness, the activity of the parasympathetic nervous system increases, which is reflected in decreased average HR (Vigo et al., 2012). The change in the structure and remodeling of the heart muscle is another clinically important effect of being in conditions of microgravity. Heart remodeling after spaceflights results in arrhythmias and various types of heart disorders, i.e., long QT syndrome and sudden cardiac arrest. Moreover, studies conducted on animal models confirm changes in the cardiac morphology. After staying in microgravity, wild-type fruit flies, *Drosophila* Canton-S, had significantly reduced expression of sarcomeric and extracellular matrix (ECM) genes and increased proteasomal gene expression, which is characteristic of proteasomal stress, myofibrillar remodeling of the heart, and its constriction (Walls et al., 2020). To minimize the effect of microgravity on the myocardium would require targeting the WW domain-containing E3 ubiquitin protein ligase 1 (WWP1), which is one of the drivers of cardiac remodeling in response to pressure overloads. A decrease in WWP1 concentration was associated with less cardiac atrophy and changes in function in response to stimulated microgravity in mice and monkeys (Zhong et al., 2021). In mice experimental model, stimulated microgravity-induced cardiac remodeling may be associated with increased phosphorylation of the cardiac ryanodine receptor (RyR2) by Ca^2+^/calmodulin-dependent protein kinase II (CaMKII), resulting in increased Ca^2+^ secretion and subsequent cardiac remodeling (Respress et al., 2014). These studies were conducted on animal models, which limits relating these research results to the physiology of the human body. Thus, human induced pluripotent stem cell-derived cardiomyocytes (hiPSC-CMs) are a new, promising approach to the study of molecular changes in the heart especially that microgravity stimulates the differentiation process of pluripotent stem cells (Li et al., 2019). Wnorowski et al. showed that hiPSC-CMs cultured for 5.5 weeks in the ISS showed reduced Ca^2+^ recycling, most probably resulting from an increase in the load on the endoplasmic reticulum and, consequently, causing an observable irregularity in the work of cardiomyocytes (Wnorowski et al., 2019).

Microgravity also factors in the reduction of left ventricular mass (LVM) of the heart after returning to Earth. In the case of short-term spaceflights, LVM decreases by 9.1%, which returns to normal within 3 days. Given similar results in dehydrated patients, these changes seem to be related to fluid replacement after returning to Earth (Summers et al., 2005). However, studies conducted on rats after a 7-day microgravity exposure suggest that a short stay in space does not affect the atrophy of the heart muscle (Ray et al., 2001).

Staying in space also changes circulation and vasculature. Unfortunately, research on long-term spaceflight is scarce. Baevsky et al. (2021) analyzed cardiac and circulatory parameters in astronauts before, during, and after a 6-month space mission, and showed, that hemodynamic proportions change significantly during a long-term stay in space. During the first month of spaceflight, a significant increase in the left ventricle activity is observed. This phenomenon may be related to acclimatization to the conditions of lower systemic blood circulation. At the same time, the pulmonary circulation filling increases, and the right ventricular systolic ejection decreases (Baevsky et al., 2021).

Another aspect of the long-term spaceflight is the microgravity effect on the arterial stiffness. Studies conducted on volunteers lying head down for a long time have shown that over time (up to 60 days) the value of arterial stiffness increases, which can be an adaptation to microgravity, but also a harmful factor for the cardiovascular system (Krachtis et al., 2022). Research carried out during a 6-month space flight also indicated a 17%–30% increase, compared to values before the flight, in arterial stiffness measured within 38 h of returning to the Earth (Hughson et al., 2016). However, it is worth noting that simulating the microgravity environment through head-down tilt (HDT) bed rest does not fully reflect the actual conditions in space, and does not reflect other processes occurring in the human body during a stay in space. But, HDT is still the most common and refined method used to study the effects of microgravity on the human body and develop preventive measures (Hargens and Vico, 2016). Other studies involving stimulation of microgravity in mice through hindlimb unloading and hindlimb reloading showed that reversing the remodeling processes of the heart is possible, but remodeling of the right ventricle is slower (Zhong et al., 2016).

## 3 Respiratory system

Mars’ atmosphere comprises 95.1% CO_2_, 2% nitrogen, and 2% argon, and is characterized by 6.36 mb (636 Pa) surface pressure and 3.71 m/s^2^ surface gravity (NASA, 2022). Conditions on Mars, the main planet considered for human settlement in the future, might have a strong influence on the human respiratory system. The major risk factors include radiation exposure, partitial gravity, changes in atmospheric pressure, and dust inhalation. Exposure to cosmic rays damages lung tissue, leading to inflammation and an increased risk of infection (Prisk, 2019). Reduced gravity can cause fluid accumulation in the lungs, leading to decreased lung capacity and breathing difficulties. Changes in atmospheric pressure lead to air bubbles formation in the lungs, causing pain and breathing difficulties. Studies on the space travel effects on the lungs indicate that lungs adapt well to the changed environment (Prisk, 2019).

Decompression sickness (DCS) is the major obstacle to the proper functioning of the respiratory system in space. DCS is indicated as one of the most important threats to humans in space, resulting from the closed environment and architecture of the space vehicle (Romero and Francisco, 2020). Henry’s Law states that the amount of a gas that can be dissolved in a liquid is proportional to the partial pressure of the gas above the liquid. Decompression sickness in astronauts can occur during too rapid reduction of pressure during extravehicular activity (EVA) preparation.

Decompression sickness affects astronauts not after, but during an EVA because the NASA spacesuit functions only at a pressure of 222 mmHg above vacuum (100% O_2_) (Powell et al., 1994; Locke, 2008), to retain astronaut mobility in the suits and limit effort during tasks execution. The pressure within the space suit is a compromise between the ability to move the limbs and DCS risk.

There are two ways to prevent DCS: 1) supplying an adequate ambient gas pressure on the body via a mechanical structure, and 2) exposing the body to a hyperbaric or hypobaric environment while dropping ambient pressure at a rate that avoids or restricts bubble formation in the tissues. The first way is more expensive regarding engineering and materials but safer. In the contrast, the second option is less expensive, but developing depressurization procedures requires understanding DCS (Conklin, 2011). Therefore, a procedure called pre-breathe was developed and improved by NASA over the years. The campout protocol is used on the ISS to reduce astronaut fatigue and boost efficiency. It is a modified shuttle routine in which two astronauts camp out at 70.3 kPa (10.2 PSI) psia in the ISS airlock for 8 h 40 min, followed by a re-pressurization to 14.7 psia once while breathing 100% O_2_ through a mask for 70 min. The phased approach is recommended over a simpler in-suit pre-breathe, because it allows astronauts to focus on other tasks rather than staying in the suit for 10–12 h. The phased procedure is preferred because the transition from a mask to a suit with 100% O_2_ without an air break might be problematic (Ross and Duncan, 2008).

Regardless of the duration of the mission, the issue of DCS remains one of the important health considerations. Nevertheless, the research suggests that the existing denitrogenation techniques in the ISS are effective at minimizing adverse lung effects (Ross and Duncan, 2008).

One of the major concerns in the context of long-term cosmic ray exposure is lung cancer. Studies in mice (Xie et al., 2020) revealed a link between cosmic ray exposure, Bcl-2 expression, and the development of lung cancer. ^56^Fe appears to be the most carcinogenic, generating squamous cell carcinoma and adenocarcinoma. Adenocarcinoma was the only result of ^28^Si and protons. It should be noted that high-energy nuclei components (HZE) of galactic cosmic rays (i.e., ^56^Fe) are a minor component of cosmic radiation. The research of heavy ion radiation effects on animals, e.g., mice provide most insight into the human health consequences of space radiation exposure, as information on human GCR exposure is limited (Weil et al., 2009; Trani et al., 2010; Suman et al., 2012; Cucinotta, 2014; Wang et al., 2015).

Space radiation leads to lung injury called SPRALI, characterized by loss of lung alveolar structures and functional alterations resulting in air space increase and diminished breathing. These long-term pathophysiological abnormalities in the lungs persist even after over 2 years of space radiation exposure and are related to abnormal cell signaling (Christofidou-Solomidou et al., 2015). Other researchers, who studied the effects of high-LET radiation on the lungs, concentrated on the possible mutagenesis consequences of radiation-induced oxidative stress or DNA damage on lung epithelial cells (Jones et al., 2007). Additional studies discovered that HZE particles leave a distinct imprint on the epigenome in human bronchial epithelial cells. Radiation-induced methylation modifications occur early and continue across time, indicating a heritable change in the epigenome (Kennedy et al., 2018). Similar changes in the epigenome of mouse hippocampal cells have been described by Impey et al. (Impey et al., 2016a; Impey et al., 2016b).

An important issue for the lungs functioning on the extraterrestial surface is the long-term exposure to dust and various inhaled particles. On the Moon, dust particles can be released by natural processes like meteoroid strikes and electrostatic lofting, or they might be stirred up by human exploration efforts (Farr et al., 2020). The harmfulness of lunar dust has been extensively described by Linnarsson et al. (Linnarsson et al., 2012). The atmosphere of Mars also contains a huge amount of suspended dust (Landis, 1998). Inhalation of particles of varied sizes may have a negative impact on the respiratory and cardiovascular systems, resulting in airway inflammation and increased respiratory and cardiovascular morbidity (Sundblad et al., 2002; Frampton et al., 2006). The risk of dust inhalation increases in a microgravity or hypogravity setting due to reduced gravity-induced sedimentation. Because inhaled particles tend to settle more peripherally, they may be kept in the lungs for longer periods in low gravity, as will be the situation in a future lunar colony (Darquenne and Prisk, 2008; Peterson et al., 2008), thus technologies reducing the problem of dust have been developed for years (Afshar-Mohajer et al., 2015).

The research on the effects of the mentioned factors on the respiratory system encompasses extended-duration missions, shorter ones, and various simulation scenarios. However, insufficient comprehensive data pertains to space missions exceeding 6 months (the average length of an ISS mission). Research on extended space missions effects is imperative, especially to evaluate potential risks that may emerge from endeavors like the journey to Mars, because making estimations based on the currently available data is beyond complex.

## 4 Nervous system

The growing interest in space tourism is the reason for numerous research on the human nervous system physiology in short and long-term spaceflights conditions (NASA, 2018).

Acute risks related to the nervous system, resulting from disrupting the connection between perception and action, include nausea, vomiting, and imbalance. Microgravity, with intermediate periods of hypergravity during launch and ascent into space and during return to the Earth’s atmosphere, as well as hypogravity during ‘walks’ on the Moon’s surface, is associated with disturbances in the vestibular system. Astronauts in the initial stages of a space mission have standard inner ear mechanisms. The state of weightlessness removes the stimulus to the otoliths, disrupting the detection of spatial orientation in response to head tilt. This may affect the vestibular nuclei and cortical projections. It is believed that as a result the central nervous system (CNS) interprets all otolith output signals as linear acceleration rather than induced by head tilt (Clément et al., 2020). It makes it impossible to determine the actual position of people and objects (Gupta et al., 2023). This hypothesis is supported by changes in astronauts’ eye movement control, posture, and gait after spaceflight (Clément et al., 2020).

Problems with the synchronization of movements observed among crew members during activities may suggest changes in cerebellar structure and function responsible for the coordination and control of fine movements (Van Ombergen et al., 2017a). This leads to dizziness, spatial disorientation, and postural instability. Adaptive mechanisms in response to short-term exposure may include temporal alteration of transduction or potentiation of synaptic conduction. Long-term exposure to microgravity may involve more complex adaptive mechanisms (Clément et al., 2020).

The vestibule-ocular reflex (VOR), which compensates for head movements, remains intact in the weightless state suggesting that gravity has no effect on the primary response of the semi-circular canals (Clément et al., 2020). Alterations in the vestibulospinal reflexes may also contribute to postural disorders. The H-reflex (Hoffmann reflex) decreases dramatically during spaceflight, due to stretching of the spinal cord, cauda equina, nerve roots, and spinal column tissue (Clément et al., 2020; Buoite et al., 2021). It has also been postulated that a potential increase in otolith mass during flight may be responsible for changes in vestibulospinal and otolith-eye reflexes (Clément et al., 2020). The vestibular system abnormalities listed above can manifest as motion sickness, spatial disorientation, delays in eye-head coordination, rotational illusion, nystagmus, dizziness, and walking difficulties (Clément et al., 2020). It has been investigated that postural disturbances occur regardless of the length of the flight. However, longer exposure leads to more severe sensory-motor disturbances, and requires longer recovery time (Clément et al., 2020; Kharlamova et al., 2021).

Space motion sickness (SMS) is one of the most distressing neurosensory phenomena for astronauts and future space tourists. It can manifest as drowsiness, vomiting, nausea, and mental and physical exhaustion (Clément et al., 2020). The severity of these adverse symptoms most likely, to some degree, depends on genetic predisposition and inter-individual differences of flight participants. Genes that may be involved in the etiopathogenesis of motion sickness are linked, among other things, to the development of the eyes (e.g., rs56100358 near PVRL3) and ears (e.g., rs12111385 near MUTED and rs1435985 near TSHZ1) (Hromatka et al., 2015). The occurrence of vestibular dysfunctions might result from fluid redistribution observed in the microgravity, resulting in pressure changes. This phenomenon may affect also the structures of the eye and brain parenchyma (Gupta et al., 2023).

Spaceflight-associated neuro-ocular syndrome (SANS), represents combinations of neuro-ocular changes caused by increased intracranial and intraocular pressure. The changes most commonly involve swelling of the optic disc, flattening of the eyeball, the appearance of choroidal and retinal folds, and foci of ischemic retinal changes (Gupta et al., 2023). Disturbances of near-field vision have also been observed as part of the neural-ocular syndrome associated with spaceflight (Gupta et al., 2023). The occurrence of the syndrome may be explained by the accumulation of cerebrospinal fluid in the extraocular space (Jillings et al., 2020; McGregor et al., 2021).

After entering the microgravity environment, the sense of sight initially takes over. This leads to the misdetermination of spatial orientation, size of objects, and optical illusions. Some astronauts report oscillopsia (illusory movement of the visual field) during motion, similar to symptoms of vagus defects (Clément et al., 2020). Occasionally, during long-duration space missions to the ISS, astronauts overestimated altitude while underestimating depth and distance. The time required to track a visual target immediately after spaceflight is also modified (Clément et al., 2020).

The mentioned changes in brain parenchymal structure due to the redistribution of body fluids were observed in astronauts and during experiments using spaceflight simulators. The observed changes in grey matter volume can be associated with decreased performance, mobility, learning, and memory abilities (Koppelmans et al., 2017; Van Ombergen et al., 2017b). Also, thinning of the cerebral cortex of the right occipital lobe and bilateral sphenoidal cortices, reduction in the size of the left thalamus, dilation of the lateral ventricle, and deformation of the pituitary gland have been noted (Arone et al., 2021).

Increased ventricular cerebrospinal fluid (CSF) volume may be associated with white matter hyperintensity (WMH), which is linked to small vessel disease in patients on Earth (Gupta et al., 2023). It has also been observed that long-term spaceflight correlates with the narrowing of the central sulcus, and CSF spaces at the vertex, and upward displacement of the brain (confirmed by ventral accumulation of free fluid) (Koppelmans et al., 2017; Roberts et al., 2017; Lee et al., 2021). Structural changes in the parenchyma and changed position of the brain may contribute to impaired CSF resorption because of the pressure on the superior sagittal sinus (Jillings et al., 2020). Long-term spaceflight may increase the risk of protein accumulation in susceptible patients resulting in premature cognitive decline. Impaired flow can also cause hydrocephalus or cerebral hydrocephalus stenosis (Gupta et al., 2023). Increased intracranial pressure (ICP) can lead to visual disturbances, nausea, vomiting, headaches, dizziness, and malaise. Changes in intracranial pressure are associated with intracerebral hemorrhage, so space travel would need to involve pre-flight cerebral vascular angiography to rule out the presence of an intracranial aneurysm (Panesar et al., 2020).

The symptoms occurring in space do include only the macroscopic changes but also involve neuroplasticity changes. Studies indicate increased functional connectivity of the motor cortex and cerebellum during spaceflight (Koppelmans et al., 2017). Van Ombergen et al. (2017a), based on spaceflight MRI studies, reported decreased functional connectivity at rest in a vestibular-related cortical area, i.e., the right insula belonging to the vestibular cortex. The results revelaed another factor underlying the motor, postural stabilisation, and spatial orientation disorders (Van Ombergen et al., 2017a). In addition, decreased cerebellar motor connectivity, between the left cerebellum and the right motor cortex, was found after the flight (Demertzi et al., 2016; Van Ombergen et al., 2017a). Neural plasticity does not only affect the cerebral cortex. Prolonged exposure to weightlessness causes anatomical and structural changes in the brainstem, hippocampus, sensorimotor cortex (Parihar et al., 2015; Gupta et al., 2023), cranial nerves, and peripheral nerves.

Cosmic radiation is one of the greatest risks to the human nervous system in space. Exposure to charged high-energy particles from galactic cosmic rays can increase the risk of neurodegenerative diseases and premature aging. Astronauts and future space tourists, especially from more susceptible groups such as children and pregnant women, are at higher risk of developing malignant brain tumors (Gupta et al., 2023). Space radiation is estimated to have much greater ionizing properties than ionizing radiation used in medicine for cancer therapy (Panesar et al., 2020; Gupta et al., 2023). Very high doses of radiation (at values higher than those to which astronauts are currently exposed) induce white matter necrosis (Panesar et al., 2020). Studies in model organisms showed that high-LET radiation and high doses of HZE ions have significant short- and long-term effects on neurogenesis in the hippocampus, inhibiting the proliferation and differentiation of precursor cells that were expected to differentiate into neurons, astrocytes, and oligodendrocytes (Clément et al., 2020; Gupta et al., 2023). LET particles can also cause capillary hemorrhages and disrupt neuroglial structure and the blood-brain barrier (Arone et al., 2021). The smaller vessels of the cerebral circulation are more susceptible to radiation damage hence capillaries are at the greatest risk. Also, in space the vessel walls thicken, narrowing the lumen and an increased risk of microaneurysms, hemorrhage, and stroke (Panesar et al., 2020). Lower doses of various HZE molecules can reduce the complexity of dendritic branches and the number of dendritic spines and associated synapses, possibly through microglia-mediated pruning, which affects processing the information in the brain (Clément et al., 2020). Marked and persistent reduction in dendritic complexity and spine density was also observed in the animals studied (Parihar et al., 2015). The observed decrease in neuromuscular response, combined with reduced gravitational load on the spine, results in flattened lumbar region, reduced range of movement, and decreased cross-sectional area of the paraspinal muscles (Swinney and Allison, 2018; Lazzari et al., 2021). NASA NEUROLAB mission results and subsequent non-invasive research suggest that during spaceflight the function of the autonomic nervous system is increased. It has been shown that baroreceptor function is impaired during spaceflight. In contrast to a vertical position on Earth, microgravity causes relative relaxation of the vessels and increased activity of the vagus nerve (Mandsager et al., 2015).

As astronauts are recruited from different cultures and nationalities, the extreme situations during space missions can lead to interpersonal conflicts and negatively affect the health and wellbeing of crew members (Gupta et al., 2023). Astronauts often experience circadian rhythm disturbances, sleep and wakefulness disorders or insomnia, which result in mood disorders, irritability, and increased stress levels related to workload. Ubiquitous sleep deprivation is a major cause of pharmacotherapy of mission participants (Putcha et al., 1999). Adequate length and quality of sleep is necessary to maintain astronauts’ wellbeing and maximum performance (Jones et al., 2022). Behavioral disorders observed during space missions and their terrestrial analogues (e.g., staying at a research station in Antarctica) may also include depressive disorders, trauma-related disorders (e.g., post-traumatic stress disorder), drug-related disorders, schizophrenia spectrum, and other psychotic disorders (Friedman and Bui, 2017). Generally, the mental state of space mission members is crucial for the course and success of the entire space mission. Transient and permanent changes in the central nervous system caused by many factors related to the space environment manifest as behavioral disorders, poor wellbeing, stress accumulation, or psychosocial disorders. Since the psychosocial aspect of long-term space missions is very complex, and includes individual, interpersonal, and organizational issues, it is necessary to implement methods that effectively control and select the candidates for space missions, especially the long-term ones (Palinkas, 2001). The initial examinations for crew members should include neuropsychological evaluation in order to stratify the risk and assess the behavioral profile of astronauts (Faerman et al., 2023). The key emotion occurring among space crew is stress. It may be mitigated in may ways, but the neurogenetic “positive approach” profile has been considered recently for astronauts. Selecting the astronauts based on biomarkers indicating better adaptation to the space environment may reduce the incidence of affective disorders and stress levels (Mammarella et al., 2022). Isolation, one of the ‘five big hazards in space,’ is an important cause of psychosocial disorders. It affects not only the mental health of the astronauts, but also interpersonal relationships among the space mission crew (Romero and Francisco, 2020).

Attachment style, interpersonal attitude, motivation, power distance, and individual-collectivism preference should also be considered when selecting astronauts and should be researched, as they are individual features (Dion, 2004). The impact of long-term space missions on the mental state and psychosocial adaptations after a space flight should also be researched (Pagnini et al., 2023). However, animal studies indicate that radiation and cosmic energy are the main cause of the central nervous system disorders and, consequently, the cause of behavioral disorders (Kiffer et al., 2019).

It is essential to pay attention to psychiatric screening when planning space travel for both qualified crew and space tourists, to disqualify candidates with pre-existing disorders, and family health history to ensure crew’s safety and the overall mission’s success.

## 5 Specialized senses

One of the most common negative effects related to vision sense observed in astronauts is the spaceflight-associated neuro-ocular syndrome (SANS). Pardon et al. reported that SANS affects around two-thirds of International Space Station (ISS) crew members (Pardon et al., 2022). The most common SANS symptoms include swelling of the optic disc, flattening of the eyeball, choroidal and retinal folding, reversible (Laurie et al., 2020) choroidal thickening, and clustering of ischemic retinal lesions (Pardon et al., 2022; Gupta et al., 2023). Pardon et al. observed increasing peripapillary total retinal thickness with concomitant macular thinning and posterior displacement of the optic disc (Pardon et al., 2022). The lesions affect both eyes and occur in both men and women (Macias et al., 2020). One of the most likely causes of SANS is cephalic fluid redistribution in a microgravity environment and associated lymphatic and vascular drainage impairment, which results in elevated intracranial and intraocular pressure, which in terrestrial conditions can be observed in patients suffering from idiopathic intracranial hypertension (IIH) (Macias et al., 2020; Lee et al., 2022). Laurie et al. suggest that fluid redistribution during spaceflight induces a chronic mild elevation of intracranial pressure (ICP) and is not pathological (Laurie et al., 2020). The chronic increase in pressure may cause mild compression, which, together with the observed choroidal thickening, may be the force responsible for compressing the macula, leading to macular thinning, which could possibly result in blurred or reduced central vision (Pardon et al., 2022). Other factors potentially contributing to SANS development include metabolic changes, inflammatory processes, axoplasmic stasis, and exposure to cosmic radiation (Wojcik et al., 2020). The syndrome is not only associated with structural changes but also translates into decreased visual comfort. Individuals affected by SANS complain of blurred close-up vision, visual scotomas, and headaches. However, no diplopia, pulsatile tinnitus, or transient visual obscuration typical of idiopathic intracranial hypertension IIH are observed. No changes in color vision have been observed (Wojcik et al., 2020; Gupta et al., 2023; Lee et al., 2022). Spaceflight conditions also cause a shortening of the axial length and anterior chamber depth (ACD) of the eye, which may be associated with astronaut’s farsightedness and correlates with the near-field visual disturbances observed in them (Lee et al., 2022; Gupta et al., 2023). No specific measures beyond *ad hoc* pharmacological measures and corrective glasses have been found to prevent the occurrence of this syndrome in the future. Therefore, it is necessary to conduct further research to establish a comprehensive program preventing or reducing the negative impact of structural changes and risk factors and improving long-term visual comfort.

Space travel is associated with deterioration and even the risk of total hearing loss, as the gravitational environment alters the way the peripheral auditory system functions and sound signals transmission and propagation. Many factors may contribute to the deterioration of hearing function: chronic exposure to noise and vibration, microgravity changes in fluid distribution, or gas levels changes at the space station like elevated carbon dioxide levels, as well as individual factors such as genetic predisposition, age, and gender (Kadem, 2018). Moreover, the elevated carbon dioxide levels increase the risk of hypoxia, which affects the functioning of other organs (Beheshti et al., 2018). Moreover, intense exposure to carbon dioxide and hearing loss are two serious threats indicated in the “big 5 hazards in space” as resulting from the specificity of space flight and spacecraft design (Romero and Francisco, 2020). Hearing tests in crew members show differences in hearing thresholds between men and women, with women presenting better results. However, no differences were observed regarding the natural age-related long-term deterioration of hearing function during spaceflight (Reschke et al., 2014). Disturbed hydrostatic equilibrium can adversely affect protective mechanisms such as the stapedial reflex and medial-olivocochlear mechanism. An increase in cochlear pressure alone can result in changes in cochlear structure and function (Moleti et al., 2019). In a spaceflight environment, astronauts are exposed to sounds between 60 and 70 dB, which combined with other factors, such as solvents, carbon monoxide, or antibiotics presence, can synergistically result in hearing loss at lower volume levels (Kadem, 2018). On the other hand, noise levels can affect hearing by affecting hair cells or ribbon synapses. Auditory thresholds are significantly reduced, and hair cell loss occurs, more severe if noise and vibration affect the individual simultaneously (Kadem, 2018). Therefore, in the era of commercial spaceflights, hearing tests seem essential for all potential space travelers. Testing must include examinations before, during, and after spaceflight to comprehensively evaluate the impact and prevent permanent hearing deterioration. Moreover, microgravity affects the function of the auditory ossicles through fluid redistribution and changes in the weight of fluid inside the ear (Kadem, 2018). The environment of weightlessness is likely to affect the auditory cortex in the temporal lobe in addition to affecting peripheral auditory structures (Kadem, 2018). The connection between hearing and balance organs suggests that the same factors may affect the sense of balance. The challenges that astronauts face include problems in determining spatial orientation, height overestimation, and objects’ depth, distance, and size reduction (Clément et al., 2020). Symptoms include fatigue, vomiting, nausea, and mental and physical exhaustion, usually occurring in the first few days of flight and upon return to the Earth’s gravity (Gupta et al., 2023). It is believed that the changes occur not only in the peripheral sensory organs but also in the cortical centers as astronauts adapt quickly while returning to space (Demertzi et al., 2016).

Since sense of smell is responsible for as much as 75%–95% of food taste perception, changes in odor perception have a significant impact on sensory impressions related to ‘taste’ in the broadest (Taylor et al., 2020). The space conditions can affect the perceived food taste. Since astronaut’s diet and food processing for spaceflight purposes are highly specific, smell and taste deficiencies might lead to insufficient food intake and development of so-called ‘space anorexia’, but due to a small research sample and scarcity of studies this hypothesis needs further evaluation (Taylor et al., 2020). Interestingly, noise and atmospheric pressure can also affect taste perception. Studies showed that white noise at 75–85 dB deteriorates sweetness and saltines perception, while low atmospheric pressure increases taste and odor thresholds (Taylor et al., 2020).

Also, extremely little data exists on cutaneous sensations under spaceflight conditions. However, it is known that the impulses for the sense of touch significantly differ under weightlessness conditions from those experienced on Earth (Buoite et al., 2021). Experiments in spaceflight simulated conditions showed that changes in somatosensory sensations and significant subjective reductions in pain sensation occur just after 2 hours of head-down bed rest (HDBR). Temperature sensation also changes under spaceflight simulated conditions. Subjects reported increased skin sensitivity to cold after 21 days of lying in bed, while after 35 days, they perceived similar temperatures as warmer and less thermally uncomfortable (Buoite et al., 2021).

More research focusing on the influence of space conditions on other receptors is still needed. Identifying potential changes in taste and smell receptors may prove to be an important factor in counteracting the incidence of anorexia among astronauts on space missions. Also, research into the effects of noise on humans in space station environment may be crucial in ensuring both physical and psychological comfort during long-duration missions.

## 6 Musculoskeletal system

The musculoskeletal system is the most one of the most extensively researched human body system in the context of spaceflights. According to “5 big hazards in space”, an altered gravitational field is closely related to both an increased risk of bone fractures and a decrease in muscle mass and strength (Romero and Francisco, 2020). Astronauts’ stamina decline is linked to mission length, individual response to altered gravity, countermeasure efficacy, and pre-mission status. Moreover, the risk of permanent disability increases proportionally as the duration of spaceflight increases (Payne et al., 2007). Based on reduced gravity tests, Laurens et al. predict that a Mars trip will result in a considerable decline in performance resulting from an estimated 15% loss of lean body mass (LBM) (Laurens et al., 2019). Loehr et al. suggested that physical preparation should begin 2 years before the flight to reduce the adverse effects of microgravity (Loehr et al., 2015), especially that Moore et al. verified that having a higher VO_2_ peak before the flight results in maintaining performance (Moore et al., 2014). There is a link between rising bone mineral density (BMD) loss and increasing mission duration, e.g., BMD measured at the calcaneus for Apollo 17 crew was 5%–6%, Skylab—4.5%–7.9%, and Salyut-6 ∼19.8%) (Rambaut et al., 1975; Stupakov et al., 1984; LeBlanc et al., 2007; Comfort et al., 2021). The loss is local (Apollo 17 crew had no changes in wrist bones compared to 5%–6% loss at calcaneus and the greatest bone mineral density (BMD) loss is observed in weight-bearing tissues like trochanter that shows BMD loss many times larger than the bones of the upper limb (LeBlanc et al., 2000).

More importantly, without exercise, the BMD loss is ten times higher than on Earth (Rambaut et al., 1975; LeBlanc et al., 2000; Berger et al., 2008), and the recovery rate is 5–6 times lower than the loss rate, particularly for extended missions (Stupakov et al., 1984; LeBlanc et al., 2007; Narici and de Boer, 2011; Hargens and Vico, 2016). Muscle loss causing loss of strength is associated with difficulties accomplishing mission tasks (Carmeliet et al., 2001; Scheuring et al., 2009; Kerstman et al., 2012; Grimm et al., 2016; Penchev et al., 2021). The loss of strength is larger than the LBM loss, and involves neurological and structural alterations in the muscles (Zange et al., 1997; Koryak, 2001; Narici and de Boer, 2011). Lean body mass and BMD loss tend to be comparable (3.5% and 3.4%, respectively for 16–28 weeks mission) (LeBlanc et al., 2000). Kozlovskaya et al. showed that spaceflight reduced maximal force production more than power output (Kozlovskaya et al., 1981). Also, Antonutto et al. observed significant losses in explosive power after 31 and 180 days of spaceflight, more significant than cycling power loss (33% and 55% vs 25%, respectively) (Antonutto et al., 1991). However, the muscle mass loss reached plateau after 9 months of simulated microgravity (di Prampero and Narici, 2003).

Space adaptation back pain (SABP) is one of the clinical musculoskeletal problems occurring during spaceflight, that is triggered by spinal elongation due to intervertebral disc enlargement (Kerstman et al., 2012; Penchev et al., 2021), traumas (Scheuring et al., 2009), bending. The workout guidelines for astronauts recommend exercising 6 days a week, including aerobic and weight activities with multiple sets (Narici and de Boer, 2011; Laurens et al., 2019) and repetitions (Hargens and Vico, 2016), and rotating the workload between days (Loehr et al., 2015; Petersen et al., 2016). However, the details vary slightly amongst space agencies. Advanced Resistive Exercise Device (ARED), Cycle Ergometer with Vibration Isolation System (CEVIS), VELO ergometer, Treadmill with Vibration Isolation System (TVIS), and T2 treadmill are among the ISS workout devices, that are used for 2–2.5 h every day (time for set-up and hygiene included) (Trigg, 2013). Advanced Resistive Exercise Device designed by NASA simulates resistance training in minimal gravity conditions by using vacuum cylinders and inertial flywheels in the structure. Research in terrestrial conditions has shown that the results of regular training using ARED do not differ from those obtained under resistance training with free weights (Lamoreaux and Landeck, 2006; Loehr et al., 2011). Aerobic exercise is also a key countermeasure, but unfortunately, treadmills are not recommended because they are unable to provide sufficient load in minimal gravity conditions. Instead, bicycle ergometers, like CEVIS and VELO ergometer, are commonly used for this reason (Thornton and Bonato, 2017). The CEVIS device allows for counteracting the vibrations that astronauts are exposed to while performing repetitive exercises in space and which affect the interference occurring during experiments (Blocker et al., 2022).

Petersen et al. found that the ISS astronauts show little or no change in BMD or aerobic capacity after spaceflight and attributed it to regular exercise and appropriate nutrient intake, including vitamin D supplementation (Petersen et al., 2016). However, earlier analyses showed that only astronauts who exercised at higher intensities and longer at higher heart rates maintained their aerobic capacity and the improvements in countermeasures reduced the muscular force decline associated with microgravity deconditioning (Moore et al., 2014). Microgravity causes bone loss through a series of mechanisms. First, microgravity triggers Ca^2+^ release from bones, which in turn suppresses the parathyroid hormone (PTH). Then, PTH suppression reduces circulating 1,25-dihydroxyvitamin D levels, leading to decreased Ca^2+^ absorption (Kerstman et al., 2012; Penchev et al., 2021). Furthermore, microgravity impairs the function of osteoblasts and increases osteocytes’ apoptosis, resulting in either unchanged or decreased bone formation and increased bone resorption, eventually leading to bone loss (Smith et al., 1999; Smith et al., 2005).

NASA does not currently recognize spaceflight-related changes in joint cartilage as a high health risk for Mars mission since it is not a direct threat to mission success. Although research on human joint cartilage modifications in spaceflight is scarce, data from cell, animal, and human studies suggests that exposure to near weightlessness mixed with radiation is likely to result in joint cartilage thinning, degeneration (Ganse et al., 2022), and eventually osteoarthritis during long-term space missions (Patel, 2020).

Space studies helped develop training techniques and pharmacological interventions preventing the bone and muscle mass loss. However, these approaches still do not offer complete control over the problem. Comprehensive data on pharmacological treatments, dose-response relationships, and the interactions between pharmacotherapy and various training modalities are still lacking. Current research primarily focuses on bisphosphonates in relation to bone health, showing a gap in the analogous studies assessing the potential impact of anabolic agents on muscles. Regarding the latter, it would be groundbreaking to determine whether it is possible to identify the substance and dosage that evoke a suitable anabolic effect while simultaneously preserving the proper function of the immune system.

## 7 Digestive system

Microgravity, cosmic rays, weightlessness, and other elements of the space environment would also greatly impact on the proper functioning of the digestive system in the long-term space missions. The incidence of digestive system illness and injury during the 180-day and 1000-day missions to Mars is 0.05 per person-year (Horneck and Comet, 2006). However, digestive disorders resulting from staying in space can cause long-term effects that threaten the health and life of astronauts. We can, among others, observe changes in the morphology of the liver leading to an early onset of non-alcoholic fatty liver disease or carcinogenesis caused by radiation (Trani et al., 2014; Vinken, 2022). As space conditions affect the function of every organ of the digestive tract it is, therefore, necessary to pay particular attention to the provision of methods to prevent and treat gastrointestinal disorders caused by exposure to space. Some of the top priority red-listed threats to humans in space in NASA’s Human Research Program include the risk of cancer and improper nutrition, which are closely related to the functioning of the digestive system during long-term space flights (Patel et al., 2020).

Changes in the abdominal organs and digestive system in long-term spaceflight conditions include enlargement of the parenchymal organs and thickening of the walls. However, these changes return to normal up to 2 weeks after the end of the mission (for those lasting up to 438 days). The severity of the described changes does not increase proportionally with the mission’s duration of up to 6 months (Afonin et al., 2003). What is more, microgravity conditions cause hemodynamics disruption which might result in the impairment of the motility of the digestive tract. Moreover, exposure to stress increases hormone levels and may be responsible for disrupting the secretory function of the glands and organs of the digestive tract (Yang et al., 2020). Some organs, like the liver, may undergo acclimation to spaceflight conditions resulting in an initial increase in bilirubin and liver-produced protein levels in the serum (Chen et al., 2020).

Saei and Barzegari (2012) suggest that research on the astronauts’ gut microbiota should be prioritized, as they are, for a long period of time, subjected to different conditions than on Earth. Space travels result in lifestyle changes, increase stress exposure, and enforce sterility of consumed meals. All those elements of space-travel induced environment could affect the microbiota and have serious implications for the astronaut’s immune system (Saei and Barzegari, 2012). The main factors affecting the microbiota composition and quantity in spaceflight conditions are microgravity, exposure to cosmic radiation, and diet changes (Siddiqui et al., 2021). MARS500 project, simulating a 520-day space stay intending to understand the characteristics of the gut microbiota in spaceflight conditions, showed that the microbiota is constantly changing, which proves that the natural properties of the microbiota have been preserved despite confined and highly regulated conditions (Turroni et al., 2017). Also, some research indicate that the intestinal microbiota may become similar among the participants possibly due to the decline in some microorganisms taxa and the adverse effects of cytokines (Voorhies et al., 2019). Turroni et al. (2017) suggested that changes in microbiota under spaceflight conditions may affect the functions of the entire organism (Turroni et al., 2017). It occurred that a 3-week psychosocial stress, which is an inherent part of a long-term space mission, decreases the number of Porphyromonadaceae in mice, especially the *Barnesiella* genus, which is one of the main representative of the intestinal microbiota in mice and humans (Alauzet et al., 2020). The decrease in the amount of gut microbiota can be prevented by providing the crew members of long-term spaceflight with a proper diet (Hao et al., 2018). Firstly, it was suggested to include appropriate amounts of fiber and energy in the diet to maintain the microbial production of short-chain fatty acids at appropriate level. Secondly, it was suggested to include probiotics and prebiotics in the diet, but further research was advocated (Turroni et al., 2020). The nutritional status of humans in space is essential for proper functioning in these conditions. Future research should focus on adapting food production methods, developing appropriate dietary practices, and selecting appropriate supplements (Tang et al., 2022).

Another important aspect in the context of astronauts’ health and functioning in the long-term space missions’ conditions is exposure to cosmic radiation, causing genetic mutations and cancers. Studies in murine models showed that exposure to radiation with high (50 cGy/min) and low (0.33 cGy/min) doses of energetically heavy ions significantly increases the incidence of gastrointestinal cancers and their grade (Trani et al., 2014). Moreover, the incidence and size of the tumor do not correlate with the dose, indicating that the energetically heavy ions (high LET) retain oncogenic potential regardless of the dose strength (Suman et al., 2017). High linear energy transfer (LET) radiation, to which astronauts are exposed, also causes metabolic disorders and radiation damage to the digestive system. The ^56^Fe-induced increase in prostanoid biosynthesis and eicosanoid signaling what affects nutrient absorption and may lead to inflammatory bowel disease, therefore these changes were suggested for biomarkers of late gastrointestinal damage after long-term space missions (Cheema et al., 2014). Studies indicate that high LET radiation affects inflammatory processes and carcinogenesis through DNA damage and chronic cellular stress. These processes are related because intestinal stem cells acquire the senescence-associated secretory phenotype (SASP). Targeting SASP may be a new therapeutic approach to preventing cancer and digestive disorders during long-term space missions (Kumar et al., 2019). On the other hand, the change in the microbiome composition in the long-term spaceflight conditions may also weaken the digestive tract defense mechanisms (Alauzet et al., 2020).

## 8 Immune system

Spaceflight conditions significantly impact the human immune system. Studies showed that astronauts present decreased number of immune cells, increased inflammation, and decreased ability to fight off infections. Additionally, astronauts may experience increased levels of oxidative stress, leading to further immune system dysfunctions (Adrian et al., 2013). These changes can be attributed to the altered gravity, radiation exposure, stress, and other factors associated with spaceflight.

Space missions’ studies showed thymus atrophy in rodents (Gridley et al., 2003; Gridley et al., 2013; Novoselova et al., 2015). Simulated rodent studies revealed increased apoptosis of double positive CD4^+^CD8^+^ (DP) precursors of differentiating T-cells in the thymus (Wei et al., 2003; Wang et al., 2007). So far, it has been demonstrated that glucocorticoids (GCs), IFN-α, sex hormones, and thymus-suppressing factors released by adipocytes can elicit thymus atrophy (Dooley and Liston, 2012). Thymic involution is a widely recognized phenomenon that includes astronauts who had lower thymic output after returning from spaceflights, most likely due to stress-induced cortisol levels (Majumdar and Nandi, 2018) and glucocorticoid activation of caspases, leading to apoptosis of thymic cells (Wang et al., 2006). Moreover, Crucian et al. reported that plasma and urine cortisol levels in ISS crew members were significantly higher (50%–200%) than in Shuttle crew members following space flight (Crucian et al., 2020). Earlier they found that general T-cell function (both CD4^+^ and CD8^+^) was reduced during the 6-month ISS spaceflight (Crucian et al., 2015). As previously mentioned, stress and its associated hormonal changes disrupt the functioning of the thymus. Further research is needed to determine if reduced gravity alone can induce similar changes.

T cells have a variety of tasks, one of which is to control the reactivation of latent herpes viruses. Most early studies focused on latent virus reactivation during short-duration spaceflight (Mehta et al., 2004; Mehta and Pierson, 2007; Mehta et al., 2013; Mehta et al., 2014). The studies focused on ISS missions (Mehta et al., 2017; Rooney et al., 2019) showed that spaceflight significantly increases the occurrence of virus reactivation. These viruses’ reactivation is an essential indicator of immunological modulation (Mehta and Pierson, 2007). Humans may be infected by eight different herpes viruses. Some of them, such as Epstein-Barr virus (EBV), have the potential to induce malignancies. Varicella-zoster virus (VZV) may produce chicken pox during the primary infection; however, VZV can reactivate later in life due to decreased immunity causing shingles (Mehta et al., 2014). Vaccination with Zostavax for VZV (Mehta et al., 2017) seems to be an effective method of preventing viral infections during long-term space flights (Spielmann et al., 2019). HSV-1 reactivation and associated skin rash have also been described (Mehta et al., 2022).

Natural killers (NK) and macrophages are also capable of causing a cytotoxic effect on abnormal, malignant, or infected cells (Adams and Hamilton, 1984; Keller et al., 1987). Many studies reported on changes in NK cell activity, reduced cytotoxicity, proliferation, and IFN-gamma secretion (Konstantinova et al., 1995; Liu et al., 2015; Bigley et al., 2019).

Long-term spaceflight missions also have a major impact on the monocytes and granulocytes receptor system involved in signal pattern recognition (Ponomarev et al., 2017). These receptors also alter many different salivary antimicrobial proteins (sAMP) (Agha et al., 2020). Several studies suggest that neutrophils and monocytes’ phagocytosis and oxidative burst capacities were significantly lower than control mean values, and also showed an increase in the number of neutrophils (Stowe et al., 1999; Kaur et al., 2004; Kaur et al., 2005).

Research on human leukocytes activated with mitogen revealed that gene expression in space and in simulated microgravity is downregulated relative to that on Earth (Hughes-Fulford et al., 2015; Martinez et al., 2015). Finally, many of the genes involved in the scavenging of reactive oxygen species were upregulated following flight, but those involved in DNA repair, protecting cells from oxidative damage, cell cycle regulation, and protein folding were downregulated (Thiel et al., 2012; Baqai et al., 2009; Barrila et al., 2016). Ionizing radiation causes chromosome-type abnormalities such as polycentric and ring chromosomes in G0 lymphocytes and DNA damage (Obe et al., 1997; Kumari et al., 2009).

A more systematic and theoretical analysis is required for the humoral response. On the one hand, there are studies showing that spaceflight and simulated microgravity can have a negative impact on B cell progenitor cells in the bone marrow, splenic B cell counts, and hematopoietic stem cell proliferation rate, which can lead to reduced Ig output in an animal model (Plett et al., 2001; Ortega et al., 2009; Lescale et al., 2015; Gridle y& Pecaut, 2016). On the other hand, some authors have recognized that astronauts’ Ig level remains at a constant level following 6 months ISS mission in relation to pre-flight values (Rykova et al., 2008). Moreover, long-duration spaceflight has minimal effect on B cell quantity, phenotypic, and antibody output in human astronauts (Spielmann et al., 2019). Buchheim et al. (2020) demonstrate that cosmonauts’ IgM repertoire differs from that of control subjects before launch and that two of the five examined cosmonauts’ IgM repertoires change over a long-term mission on the ISS (Buchheim et al., 2020). A wide spectrum of biochemical markers (cytokines and chemokines) associated with inflammation, cell growth, and proliferation, as well as tumor proliferation and vascularization, increased during flight and considerably decreased upon return (Vallet et al., 2011; Amarasekara et al., 2018; Yano et al., 2005; Garret-Bakelman et al., 2019).

General research shows gender-specific differences in immune responses, with women expressing a generally elevated immune response and a higher incidence of autoimmune disease compared to men. Menstrual cycle can also affect type1/type2 cytokine balance and immunological reaction (Agarwal and Marshall, 1999; Harm et al., 2001), and spaceflight conditions seem to trigger T helper 2 (Th2) shift in some cases (Crucian et al., 2008). Cytokine imbalance may result in skin reactions and rhinitis (Crucian et al., 2016). Furthermore, thymic dysfunction may be linked to an increased risk of autoimmunity due to enhanced homeostatic proliferation or loss of regulatory T cells after lymphocyte reduction (Sakaguchi et al., 1995; Jones et al., 2013; Benjamin et al., 2016). It seems reasonable that before any extensive study of in-flight gender-based responses is undertaken, additional gender differences correlating robust immunological tests and confirmed clinical outcomes must be performed by Earth-based research.

In August 1972, radio blackouts and TV interference caused by a solar storm (Omatola and Okeme, 2012) widespread the knowledge of cosmic rays. Cosmic rays effects on human health in the context of long-term space missions have been researched extensively, especially carcinogenesis. The demand for astronaut radiation protection increases in long-term spaceflight outside the Earth’s magnetosphere conditions. In 2018, NASA created a GCR simulator for radiobiological research (Simonsen et al., 2020). The majority of current research points to gene mutations and chromosomal abnormalities caused by DNA damage, due to reactive oxygen species and epigenetic changes as the primary mechanisms, enhanced by radiation exposure increasing the frequency of malignancies and hereditary consequences (Hanahan and Weinberg, 2011; United Nations, 2023a). NASA researched how space radiation exposure modifies the key genetic and epigenetic changes—the hallmarks of cancer, in order to compare the risk of HZE tumors to either radiogenic tumors induced by ground-based radiation or spontaneous tumors. Malignancies of epithelial origin, especially the lung, breast, stomach, colon, and bladder cancers, and leukemias are of the principal concern (Patel et al., 2020). Several studies suggest that proton irradiation-induced DNA damage was more severe and acute, as resulted in more premalignant lesions, and the foci identified were larger than in control and gamma radiation-exposed mice (Gerelchuluun et al., 2011; Luitel et al., 2018). Additionally, space conditions seem to enhance genome instability expressed as increased frequencies of chromosomal inversions during and after spaceflight (Luxton et al., 2020). The genetic youth associated with telomere lengthening in space is only apparent. Research on the telomeres length, important in the malignant transformation of some cancers (Zhu et al., 2016), showed that telomeres were longer during spaceflight, and upon return to Earth shortened rapidly, resulting in many more short telomeres after spaceflight than before (Luxton and Bailey, 2021). Regrettably, the lack of properly simulated conditions and the restrictions for research on animal models make the analysis of the high-energy and low-dose-rate radiation effects very difficult. There are significant gaps in our understanding of the impact of space radiation on human tumor morbidity and death.

The immune system seems one of the most extensively clarified systems in scientific literature. Inquiries into how space missions impact humoral responses and how cosmic radiation relates to processes culminating in cancer and cancer-related mortality should be made. Research data show that passive protective measures against cosmic radiation prove inadequate and cannot be used as a sole countermeasure ensuring safety during Mars missions. Hence, the concept of active safeguarding demands further progress.

## 9 Excretory and reproductive systems

The complexity of the human endocrine system, inter-individual and gender differences, differences in secretion frequency and intensity, and the multifaceted relationships between hormones pose significant difficulties for researchers investigating the spaceflight effects on the human endocrine system.

One of the phenomena adversely affecting human health observed during flight is the already described here bone mineral density loss. The process of bone mass loss which may also result from hormonal changes under space conditions. PTH produced by the parathyroid glands, promotes osteoclast activity, influences Ca^2+^ resorption from bone and from the urine and its absorption from the gut. Some studies in animal models and some human studies in simulated spaceflight conditions indicate elevated PTH values, while others describe no such changes (Bikle et al., 1997) or even PTH decrease (Strollo, 1999). The vitamin D3 active form produced by the kidneys increases Ca^2+^ absorption in the intestines and its resorption from the bones. It has been observed that vitamin D3 active form concentration increases significantly during the first days of spaceflight, which may suggest that it may initiate Ca^2+^ loss from bones (Leach et al., 1988; Strollo, 1999). Hirayama et al. (2023) highlight that bone metabolism might be controlled by melatonin through its effects on thyroid and parathyroid cells, which control calcitonin and PTH-mediated bone demineralization (Hirayama et al., 2023). Calcitonin produced by parafollicular thyroid cells (C cells) enhances osteoblasts and inhibits osteoclasts activity (Strollo, 1999). Animal studies researching the impact of astronauts’ isolation reported changes, associated with loss of C cells, in the thyroid gland structure, resulting in reduced calcitonin production (Albi et al., 2014). Studies on thyroid function showed that spaceflight conditions can induce mild hypothyroidism with compensatory pituitary hyperactivity (manifesting with elevated TSH levels during flight) both in humans and animals (Strollo, 1999; Albi et al., 2014).

Spaceflight conditions may also influence hypoandrogenization that exacerbates bone demineralization (Strollo, 1999). Long-term spaceflight as a source of constant physical and psychological stressors increased the hypothalamic-pituitary-adrenal (HPA) axis activity in astronauts, which was associated with decreased immune system activity and increased bone loss (Angeloni and Demonstis, 2020). However, earlier studies showed no increase in cortisol in astronauts’ plasma under simulated weightlessness conditions (Leach et al., 1988) and later it was suggested, that spaceflight is not associated with significant changes in hypothalamic-pituitary-adrenal axis activity (Stein et al., 1999).

Studies conducted over the years in space suggest that the microgravity conditions induce subclinical diabetic changes, resulting in bone loss and muscle atrophy. The observed changes in insulin secretion and sensitivity, glucose tolerance, and protein and amino acid metabolism support the hypothesis that insulin plays an essential role in maintaining muscle mass during long-term spaceflights (Tobin et al., 2002). Changes in insulin production and sensitivity may be important factors behind protein loss. Stein et al. linked insulin resistance to a nervous tension state, imposing constant threat when entering and staying in space (Stein et al., 1999). The researchers suggest that, the secretory activity of both extra- and endocrine pancreatic cells decreases in the microgravity environment. However, these deviations return to average values during the readaptation period (Proshchina et al., 2015). The reduced under microgravity conditions insulin and glucagon production resulted in their inhibited secretion during the first hours after landing. The insulin and glucagon metabolism was restored to normal levels 48 h after landing (Proshchina et al., 2015). Other studies reported increased secretion of insulin and pancreatic digestive enzymes and incompatible C-peptide values in the blood of astronauts in the early post-space period (Proshchina et al., 2015). Hughson et al. reported an inflight insulin resistance during a 6-month space flight (Hughson et al., 2016).

Recent studies show that the microgravity conditions induce a decrease in growth hormone (GH), adrenocorticotropic hormone (ACTH), and norepinephrine levels (Hirayama et al., 2023). The measured, in urine samples, decreases in triiodothyronine and norepinephrine secretion were small, early and late in the mission. However, after landing these values increased and exceeded those noted before the flight (Hirayama et al., 2023).

Harmful effects of galactic radiation are also associated with the increased risk of abnormalities in male and female reproductive systems. The reproductive cells secreted in the ovaries and testes are extremely susceptible to radiation and mutation processes.

Studies found, among other things, impaired spermatogenesis (Strollo, 1999; Ahrari et al., 2022), reduction in the total number of sperm cells and their motility, and sperm cells DNA fragmentation (Ahrari et al., 2022). At the hormonal level, both actual and simulated spaceflight conditions inhibit androgen production. Testosterone levels decrease, resulting in hypogonadism (Mishra et al., 2016; Ahrari et al., 2022). Serum levels of luteinizing hormone (LH) increase, which is associated with reduced negative testicular feedback (Mishra et al., 2016). However, some studies contradict these findings, but the differences may result from different exposure time, astronauts age, or psychological aspects, factors that impact the physiology of the reproductive system (Smith et al., 2012). Studies on rat fetuses investigating the effects of space showed no change in the number of healthy and atretic ovarian antral follicle populations, *in utero* fetal abnormalities, plasma progesterone and luteinizing hormone (LH) concentrations, or pituitary follicle-stimulating hormone (FSH) content. However, in *postpartum* samples the researchers observed an increase in plasma FSH levels and a decrease in pituitary LH content. None of the ovarian parameters were altered by spaceflight conditions (Burden et al., 1997). Rose. (2022) warn that a typical spaceflight to Mars could reduce women’s ovarian reserve by up to 50%, which may be associated with the premature menopause occurrence, and therefore the risk of possible premature mortality (Rose, 2022). Mishra et al. (2016) reported that female astronauts tend to postpone possible pregnancy and suppress menstrual cycles during spaceflight with oral contraceptives containing estrogen and progestogen, which may result in an increased risk of possible unwanted infertility (Mishra et al., 2016). To prevent this, the female astronauts might subject to estrogen replacement therapy or oocyte cryopreservation and ovarian cortical tissue freezing (Rose, 2022).

Due to ethical controversies and the lack of clear evidence on the direct impact of space travel, some studies have been conducted on animals. Iriji et al. (1998) proved that animals fertility and reproductive capacity in space are species-dependent. Comparing amphipods, pond snails, ostracods, and daphnia proved that daphnia is the most sensitive invertebrate among the tested. However, all of them survived and produced offspring by repeating their life cycles during the 4-month stay in space. However, Ijiri et al. suggested that microgravity in space may impact the processes occurring during embryogenesis as some amphipods offspring had abnormally developed limbs (Ijiri et al., 1998). Animals are also used on the ISS to obtain gametes for analyzing the damage done by cosmic rays that are nearly 100 times more powerful than on Earth and damage the genetic material which can affect fertility. Wakayama et al. analyzed mouse spermatozoa that were freeze-dried and stored in the ISS for 9 months at −95°C and they observed the evidence of DNA damage, but no differences in birth and conception rates. In addition, the offspring obtained from these gametes did not show signs of infertility (Wakayama et al., 2017). Moreover, their recent studies indicate that the freeze-dried mouse sperm is still viable after 5 years of storage in a space station and continuous exposure to cosmic radiation. The offspring obtained from these gametes is no different from the offspring obtained from gametes stored under normal conditions. Wakayama et al. suggest that freeze-dried sperm could be stored in the ISS without protection against cosmic radiation for over 200 years (Wakayama et al., 2021). It is believed that the influence of cosmic rays is the most dangerous for astronauts reproduction and fertility (Barbrow, 2020). One of the components of cosmic radiation is charged iron particles - LET radiation, which, occurring alone, causes premature ovarian failure in mice even at low doses, and negatively affects hormonal balance, fertility, and other organs (Mishra et al., 2016). Additionally, Qin et al. noticed that short-term exposure to microgravity did not cause serious changes in the reproductive organs, but the 24-h exposure to cyclic ionizing radiation caused noticeable changes in the tested mice. They noted decreased activity of testicular enzymes and sperm motility with their increased production (Qin et al., 2021).

In recent years, a new branch of human health aspects in the context of long-term space missions has been noticed - space sexology. Taking into account the growing number of spaceflights as well as considerations about human life in space and on planets other than Earth, it seems important to pay attention to human need for intimacy and, resulting from the need, sexual expression in space. The development of this research topic seems important due to the specificity of the environment in which astronauts live: limited privacy and access to partners, sexual abstinence, and hygiene rules (Dubé et al., 2023). Gender differences are another very important aspect of the demographics of space mission participants (Ronca et al., 2014). Comparing data on conceptions, births, and child status of US astronauts and comparing them with data on birth demographics in the US, it is hypothesized that short-term spaceflights (up to 9 days) do not significantly affect astronauts’ reproductive capacity (Jones et al., 2005). Similar conclusions were drawn comparing rats spending 35 days on the ISS and on Earth: their gonads did not differ in structure or genetic defects. The sperm of rats placed in the ISS was able to fertilize at a similar level to the sperm of the control sample (Matsumura et al., 2019).

Despite the low effect on the fertility of short-term space missions, the current problem is to investigate the effect of long-term space missions on human reproductive capacity. Microgravity, cosmic radiation, and weightlessness are the main factors that disturb the homeostasis of the organism in space. Thus, the study of the chronic effects of these factors on reproduction is necessary for the progress of space technology, the increasing duration of space missions, and plans to colonize other planets such as Mars. *In vitro* studies with artificially induced microgravity stimulation indicate that with long-term exposure, microgravity does not affect follicular survival and growth but reduces the quality of oocytes released from cultured follicles, as measured by the number of growth factors secreted by them, such as growth differentiation factor 9 (GDF9) (Cheng et al., 2023). Studies conducted on mice placed in the space station for 91 days showed that due to the exposure, the concentration of steroid dehydrogenase 3b and 17b decreased, and the sperm count was reduced by 90% compared to the control group. Notably, the tubular architecture of the seminiferous tubules has also changed, which may indicate the influence of long-term stay in space on the appearance of degenerative changes in the reproductive organs (Masini et al., 2012).

Another important aspect is the issue of the impact of microgravity on the release, the concentration of sex hormones, and the regulation of hormonal axes during a stay in space conditions. In rats, during a 14-day stay in space, the amount of luteinizing hormone and testosterone excreted in the urine increases during the first 3 days after returning to Earth, which indicates the adaptation of the pituitary-gonadal axis to stay in microgravity (Ortiz et al., 2000). Earlier studies conducted on rats showed that during a 14-day spaceflight, the quality of the spermatogenesis process did not change, but the level of testosterone in the blood decreased significantly (Amann et al., 1992). In mice, during a 31-day spaceflight, the amount of progesterone secreted by the ovaries decreased, while estrogen levels remained unchanged. During a flight of this length, the level of expression of genes involved in ovarian steroidogenesis did not change either (Hong et al., 2021). Studies on pregnant mice placed on a space shuttle for 11 days showed that the flight completed during pregnancy and ended before birth did not affect either the weight or hormonal parameters of the ovaries. The only effect of spaceflight was noticed for pituitary hormones, as plasma FSH level increased and pituitary LH level decreased (Burden et al., 1997). The study was conducted in a short-term spaceflight conditions, but taking into account the length of pregnancy in rats (21–23 days) the 11-day stay in space was half the duration of rats pregnancy.

It would be interesting to check how the proportional stay in space affects the development of the human fetus and the function of the female reproductive system. It has been found that embryos can develop in space. The genome of blastocysts developed under cosmic radiation exposure is characterized by significant genetic damage and epigenetic changes in the form of global hypermethylation with differentially methylated regions (DMRs). DMRs are associated with key processes for proper embryonic development, regulation of RNA metabolic processes, and regulation of intracellular protein transport (Lei et al., 2020). Another factor that may adversely affect fertility and reproduction is oxidative stress to which astronauts are exposed. The pro-oxidation state is related to the interaction of cosmic rays and microgravity. The observed oxidative stress of the placenta is an unfavorable prognostic factor for the developing fetus, hence the interaction of these three factors (cosmic rays, microgravity, and oxidative stress) may increase the risk of miscarriages, premature births, and pregnancy-related complications such as pre-eclampsia or gestational diabetes (Steller et al., 2018).

There are still many gaps in the current knowledge about the impact of long-term space missions on the functioning of the human reproductive system. Much is known about the impact of microgravity and cosmic radiation on gametes and reproductive cells. It is certain that the space environment is not without influence on the human reproductive system. Future research should focus on examining the influence of the space environment on the hormonal regulation of reproduction, and prenatal development of the reproductive system. At the same time, research conducted both in the ISS and in stimulated space conditions on Earth should focus on minimizing and neutralizing damage to genetic material, cells, tissues, and organs (Mishra and Luderer, 2019). Other factors affecting the risk of reproductive life in space, such as oxidative stress and cellular stress, should also be taken into account. The influence of short-term spaceflights on reproduction is particularly well presented in the literature, especially the studies on animals. Therefore, using the knowledge gained through research on animal models, the impact of long-term space flights, and the risk factors associated with them on human cells and genitals should be explored more extensively. Taking into account the previous reports on the harmfulness of microgravity and cosmic radiation, these studies should be carried out under special rigor and supervision in the field of research ethics.

## 10 Summary

According to the previously mentioned “5 hazards in space” theory, the success and course of a space flight and the degree of flight impact on the human body depend primarily on the five threats to which astronauts are exposed. Additionally to changed gravity and cosmic radiation, isolation is considered an important threat affecting the behavioral profile and wellbeing of crew members. The two remaining threats: dependence on spacecraft and distance to Earth require intensive research to ensure safety and security of space missions (Romero and Francisco, 2020).

The increased interest in space travel requires continued research into its effects on humans. Scientists emphasize that achieving a complete understanding of the effects of being in space on humans will require a series of experiments and extensive scientific, research, and engineering collaboration in specially created training centers and space medicine hospitals. At the same time, the greater availability of extraterrestrial voyages will make the research group significantly larger. However, before this can happen, a great deal of emphasis must be placed on screening to eliminate the risks known to us now.

Conquering space is a great and difficult challenge, but there is no shortage of volunteers willing to take it on, despite the many documented risks. With great determination, they aim to explore space as a place where humans could settle, as well as a place to obtain new raw materials. Therefore, in recent years, space exploration has become highly fashionable for international and private investors. Hence, much effort and attention is directed toward understanding how a prolonged stay in space affects the human body and psyche. It is essential to create comprehensive, individualized nutritional strategies and exercise regimens for astronauts to protect them from harmful metabolic changes that can result in the multi-organ changes described above.

Understanding and developing methods to offset the adverse effects of prolonged stays outside Earth orbit on the human body is a priority task, especially given the development of space tourism. The countermeasures eliminating the harmful effects of prolonged weightlessness on the human body must be improved. Further research (Table 1) and the development of space medicine are essential if the dream of galactic colonization is to become a reality.

**TABLE 1:** Directions of future research on the impact of long-term space flights on the human body.

Human body system	Research direction
All systems	Creating an artificial gravity station whose conditions would faithfully reproduce terrestial conditions, which would expand knowledge on human physiology in space
Respiratory system	Determining how exposure to cosmic radiation affects the human respiratory system, and in particular what is the specific relationship between such exposure and the potential development of cancer in astronauts
Nervous system	Investigating the impact of exploration, meaning specific stressors, including isolation and confinement, on the wellbeing, cognitive function, and immune health of crew members
	Designing space station for greater physical and psychological comfort: reducing noise and vibration, adequate lighting to benefit the circadian rhythm
	Developing emergency management schemes for neurological diseases and psychiatric emergencies in space
Specialized senses	Identifying potential changes in the perception of taste and smell as a means of preventing anorexia in space
Musculoskeletal system	Determining the relationship between baseline bone mineral density (BMD), muscle strength, and muscle mass Determining the condition of the musculoskeletal system after spaceflight, and checking whether higher initial physical fitness has a positive effect on regeneration after long-term spaceflight
	Exploring the potential use of eccentric training as a supplementary method to current resistance training practices in space, and how it builds upon the effects of higher-load resistance training
Excretory system	Developing standardized experiments to determine changes in hormone fluctuations and their effect on humans as data on hormone levels in space are often contradictory
Reproductive system	Investigating the effects of space conditions on fetuses and children development in the perspective of life in space
